# Multiplexed In Situ Spatial Protein Profiling in the Pursuit of Precision Immuno-Oncology for Patients with Breast Cancer

**DOI:** 10.3390/cancers14194885

**Published:** 2022-10-06

**Authors:** Davide Massa, Anna Tosi, Antonio Rosato, Valentina Guarneri, Maria Vittoria Dieci

**Affiliations:** 1 Department of Surgery, Oncology and Gastroenterology, University of Padova, 35128 Padova, Italy; 2 Division of Oncology 2, Istituto Oncologico Veneto IRCCS, 35128 Padova, Italy; 3 Immunology and Molecular Oncology Diagnostics, Istituto Oncologico Veneto IRCCS, 35128 Padova, Italy

**Keywords:** breast cancer, multiplex, spatial profiling, TILs, spatial biology, cancer-immunity cycle

## Abstract

**Simple Summary:**

Immune checkpoint inhibitors (ICIs) aim to re-establish cancer immune control by modulating immune-inhibitory signaling pathways. ICIs are currently approved in breast cancer treatment act by blocking cell anti-PD-1/PD-L1 interactions. Nonetheless, as many mechanisms of immune escape can underlie the insurgence of cancer cells, most patients progress to ICIs, even when combined with chemotherapy. Multiplexed single-cell spatially resolved tissue analysis, by combining monoclonal antibodies with different reporters, can obtain precise single-cell epitope colocalization and thus allow to infer cellular functional states, while conserving their spatial coordinates. In this review, we highlight the potential of this technology in the context of breast cancer by selecting relevant prognostic and predictive markers through the lens of the cancer-immunity cycle.

**Abstract:**

Immune checkpoint inhibitors (ICIs) have revolutionized the treatment of many solid tumors. In breast cancer (BC), immunotherapy is currently approved in combination with chemotherapy, albeit only in triple-negative breast cancer. Unfortunately, most patients only derive limited benefit from ICIs, progressing either upfront or after an initial response. Therapeutics must engage with a heterogeneous network of complex stromal–cancer interactions that can fail at imposing cancer immune control in multiple domains, such as in the genomic, epigenomic, transcriptomic, proteomic, and metabolomic domains. To overcome these types of heterogeneous resistance phenotypes, several combinatorial strategies are underway. Still, they can be predicted to be effective only in the subgroups of patients in which those specific resistance mechanisms are effectively in place. As single biomarker predictive performances are necessarily suboptimal at capturing the complexity of this articulate network, precision immune-oncology calls for multi-omics tumor microenvironment profiling in order to identify unique predictive patterns and to proactively tailor combinatorial treatments. Multiplexed single-cell spatially resolved tissue analysis, through precise epitope colocalization, allows one to infer cellular functional states in view of their spatial organization. In this review, we discuss—through the lens of the cancer-immunity cycle—selected, established, and emerging markers that may be evaluated in multiplexed spatial protein panels to help identify prognostic and predictive patterns in BC.

## 1. Introduction

Uncontrolled cancer cell growth is fueled by genetic mutations, epigenetic modification, and post-transcriptional alterations that govern the acquisition of some key traits, which are named the hallmarks of cancer [1]. The detection of similar phenotypes across different tumors can be viewed as an expression of convergent evolution, joint solutions toward common selective pressures. The activity of the immune system, the main guardian against this opportunistic growth, can be rationalized through the framework of the cancer-immunity cycle [2,3]. This cycle is a continuous succession of events that starts with the recognition of cancer antigens by the innate immune system, a necessary step in order to prime T cells. This encounter can then be followed by a rapid expansion of T cells, which, looking for their cognate antigen, infiltrate the tumor microenvironment (TME) in order to find and destroy cancer cells. Unfortunately, each cancer cell can develop multiple strategies to circumvent their killing thanks to an extensive and plastic rewiring of their transcriptomic, genomic, proteomic, and metabolomic phenotypes [1,4], thus molding the TME to their needs. In addition, cancer cells can dampen the immunogenicity of T cells by restraining their antigenicity and limiting the release of danger signals that would otherwise alarm and activate the immune system [5]. Finally, by remodulating the TME arrangement and composition, cancer cells can induce an immune-suppressive functional status in stromal cells and therefore limit T cell entry and killing proficiency. This complex and varied ecosystem [6] of multi-layered interactions between cancer and stromal cells is the cornerstone of the profound heterogeneity of cancer and also a significant driver of their therapy resistance [7].

Breast cancer (BC) constitutes the most frequently diagnosed cancer and, among women, the global leading cause of cancer death [8]. Despite the numerous advances in treatment strategies, metastatic breast cancer (mBC) remains an incurable disease, irrespective of subtype, as most patients become resistant to even the most effective treatments [9]. In BC, immune checkpoint inhibitors (ICIs) are approved for triple-negative breast cancer (TNBC), in the advanced setting (mTNBC) for PD-L1+ patients [10,11], and in the high-risk early stage setting (eTNBC) as part of the neoadjuvant treatment [12]. Although a subgroup of patients can achieve long-lasting responses, there is still much room for improvement regarding the efficacy of this strategy, as most patients with mTNBC relapse either upfront or after an initial response, while being exposed to potentially fatal immune-related adverse events. In eTNBC, the addition of anti-PD1/PD-L1s improves the pathological complete response (pCR) rates [13] and event-free survival (EFS [12,14]; still, one-third of patients treated with pembrolizumab who do not achieve a pCR, and even a few patients who do achieve a pCR, will relapse [12].

Precision immune-oncology calls for the dynamic identification of sensitivity and resistance biomarkers in order to tailor treatments [15,16]. In fact, to reestablish cancer control, therapies must engage with a complex set of adaptations, either already employed or subsequently put in place [17], which can induce a failure in the immune system at several steps [18]. Thus, the performance of a single biomarker is largely inadequate to portray this complex network [15]. In fact, even though PD-L1 immunohistochemical (IHC) expression provided predictive value over the benefit of ICIs in treating mTNBC, its evaluation has been flawed by both analytical and conceptual limitations [19,20,21], as most PD-L1 positive patients will still progress, while some PD-L1 negative patients can still benefit from anti-PD-L1/PD-1 treatment. Further, paradoxically, PD-L1 expression evaluated in the neoadjuvant setting is not predictive of pembrolizumab benefit, being instead correlated with increased pCR rates both in CT/placebo and in the CT/pembrolizumab arms [12]. Moreover, as both epithelial and stromal cells are involved in the perturbation induced by the blockade of the PD-1/PD-L1 axis, and as both can express PD-L1 [22,23], it is still unclear, to date, which cells are the main target of ICIs.

Appraising the density of tumor-infiltrating lymphocytes (TILs) has an established prognostic role in BC, particularly within TNBC [24], and some studies suggest that increased TILs may be associated with sensitivity to ICIs [14,23,25,26]. Tumor mutational burden (TMB) and microsatellite instability status (MSI), which are indirect measures of tumor antigenicity, have provided some encouraging data in predicting the benefit of ICIs in treating eTNBC [14], mTNBC [26,27], and HER2+ BC [28]. However, the incidence of TMB-high and MSI-high statuses in BC is low [29,30] and their evaluation can provide some contradictory results [31,32]; this is because many subsequent steps in cancer recognition and killing can be subverted, despite the production of tumoral neoantigens. Composite bidimensional scores, by combining biological information from different biomarkers—such as TILs subpopulations [33], PD-L1, TMB, and gene-expression profiling (GEP)—have already provided more informative data [34,35]. Still, many of those markers correlate with each other [33,36] and only partially recapitulate the complexity of the immune contexture.

Spatially unresolved single-cell omics have allowed oncologists to define numerous atlases of TME-cell states and to identify conserved cellular phenotypes across histologies [37,38,39,40,41,42]. Nevertheless, more data are adding up on the determinant role of the spatial context as a critical determinant of each cell’s functional status [37,38,43,44]. In fact, cells, by interacting with their surroundings [45], adapt to different niches and thus acquire unique plastic phenotypic traits [46], which can influence prognosis [47] and treatment-resistance [48]. 

Multiplexed single-cell spatially resolved tissue analysis has the potential of computing different cell states, densities, positions, interactions, and aggregation in multicellular modules [49,50], and thus infer their functional status, while preserving TME architecture [51,52]. Spatial protein profiling in cancer tissues is based on the recognition and colocalization analyses of multiple epitopes, thereby achieved by combining antibodies (Ab) conjugated with various reporters. Among spatially resolved tissue analysis methods, this technique is, up to date, the one with the broadest diffusion as a research platform in BC, as recently reviewed [53]. Various workflows are in development in order to overcome the analytical [54] and computational [55] challenges related to the interpretation of this high-throughput data. Nevertheless, multiplexed spatial profiling, when using just 2–3 parameters (such as in T cell activation states, density, and PD-1/PD-L1 proximity) has already been demonstrated to be superior to the combined evaluation of TMB, PD-L1, HLA, and GEP in predicting anti-PD-1/PD-L1 benefits in other cancer types [56]. However, even though multiplex proteomic analyses can help us to study the coexpression of multiple biomarkers in situ, not all the TME components can be easily detected by immunohistochemistry, such as intracellular cytokines, which are indicative of the functional status of the tumor milieu. Moreover, a more comprehensive characterization of the activation or inhibition of specific molecular pathways is particularly informative about the tumor immune response. For these reasons, techniques have recently emerged that combine tissue biomarker characterizations and in situ transcriptional profiling, thus achieving a more extensive overview of TME [53].

As multiple combinatorial treatments are constantly brought to the clinic [16], TME-spatial profiling could constitute a context-dependent platform to integrate multi-omics cell technologies [57,58], thus enhancing the identification of potential hallmarks of therapy resistance, tailor treatments in advance, and maximizing responses, while also de-escalating unnecessary treatments.

In this review, we discuss selected, established, and emerging markers that characterize the tumor–immune interplay and that may be combined in multiplexed spatial protein profiling in order to define prognostic and predictive patterns in BC. This is not intended as a comprehensive overview including all the markers of interest, but rather a framework to rationalize the potential applications of this technology, through the lens of the cancer-immunity cycle.

## 2. Antigenicity

### 2.1. Tumor-Specific Antigens

The continuous acquisition of mutations, fueled by genomic instability, drives the collection of enabling characteristics for cancer development and progression [59]. A byproduct of this process is the formation of neoepitopes, called tumor-specific antigens (TSAs), which can be targeted by the immune system to establish cancer control. This potential of recognition constitutes the rationale for the clinical activity of ICIs in TMB-high and MSI-H cancers. Nevertheless, in BC patients with low CD8+ T cells, the evaluation of the TMB is not only uninformative of ICI benefit, but can even be associated with ICI-resistance [32], as the antigens produced by cancer cells must be somehow presented to the immune system to elicit an immune response. The complex system responsible for this task, the antigen-presenting machinery (APM), is composed of many proteins, such as the transporters associated with antigen processing (TAP) 1–2 and major histocompatibility complexes (MHCs) class I-II [60]. Cancer cells may escape antigen recognition by T cells via alterations in the APM functionality [61], which can be achieved through mutations or loss of function in key APM [62], as well as potentially reversible transcriptional [63,64,65], epigenetic [66] or post-translational modulations [67,68]. Many of these deregulations can converge into a reduced MHC-I (HLA-I) expression on the cancer cell surface. In this regard, any single-omic biomarker profiling would necessarily underestimate the degree of interference in APM efficiency [62] and therefore lose the opportunity to predict therapy resistance and implement different approaches to revert them. For example, multi-omic characterization of the TNBC mesenchymal subtype (as defined by gene expression), despite being characterized by high TMB and genomic instability, shows low proteomic and transcriptional expression of antigen-presenting genes, an absent infiltration of immune cells, and low expression of PD-L1 [66]. In this TNBC subtype, MHC-I downregulation may be mediated by a polycomb repressor complex 2 (PRC2)-mediated epigenetic silencing, which could be reverted by a PRC2 inhibitor [66].

Those strategies are highly effective immune escape mechanisms, as cancers that downregulate HLA-I tend to have a lower density of TILs [47,69,70,71] and are generally more resistant to ICIs [70]. Moreover, in patients with HER2+ BC, high HLA-I expression correlates with an excellent disease-free survival (DFS), irrespective of a pCR [72]; consistently, the coexistence of high-TILs and high-HLA-I expression is associated with an excellent prognosis when compared with patients with high-TILs, but low-HLA-I expression [73]. Nevertheless, cancers with extensive immune infiltration can still downregulate HLA-I expression as an acquired mechanism of resistance [74], further underlining the potential role of including HLA-I expression in biomarker panels. Intriguingly, in HR+/HER2− BC, high HLA-I expression is associated with higher pCR rates and infiltration of TILs but worse DFS and OS [71].

As MHC class I act as a coinhibitory receptor for natural killer (NK)-mediated killing, its loss exposes cells to NK-mediated killing. Consistently, patients with HER2+ BC with HLA-low/normal expression, but a high infiltration of NK cells, have an excellent DFS [72].

MHC-II are complexes of antigen-presenting proteins (HLA-DR, DQ, and DP) generally expressed on the surface of APCs and whose spatial interaction with T cells is essential in order to mount an effective immune response [75]. MHC class II expression in cancer cells, in many cases a byproduct of effective IFN-gamma signaling [76], correlates with a better prognosis [77] and is a positive predictive factor of ICI response in many cancer types [76,78,79,80,81], including BC [82,83]. Intriguingly, as ER-signaling can directly impair MHC class II expression [84], HLA-DR expression is infrequent in HR+/HER2− BC [71], thus possibly contributing to the reduced immunogenicity of this BC subtype [85]. Despite this, MHC-II expression is a potent positive predictor of benefit from an anti PD1/PD-L1 addition in the neoadjuvant setting in both TNBC [82,83] and HR+/HER2− BC patients [82]. Interestingly, this positive correlation could be related to the firm reliance of MHC-II + cancer cells on immune checkpoint expression (such as PD-L1) as protection against CD8+ T cell-mediated killing [86]. This dependency on PD-L1 expression could result from the direct interaction between CD4+ T cells and MHC-II [87], which can either reinforce the cell-killing activity of CD8+ T cells that are already engaged by MHC-I or directly kill cancer cells [88,89]. In fact, in TNBC, an MHC-II positive phenotype is associated with a high infiltration of TILs [69,90,91], CD4+ T cells [86], and a higher expression of PD-L1 [82,92]. Interestingly, this sensitivity to ICI may be matched by a unique set of immune escape mechanisms, such as a high infiltration of TILS expressing LAG-3+ and FCRL6+ that can interfere with MHC-II antigen presentation, thus disrupting the interaction with CD4+ T cells. As such, this acquired escape process may limit the predictive value of MHC II as a single biomarker in this context [86].

Combined bulk profiling of proteins that are related to MHC I and II, such as TAP1 and HLA-DQ, can identify BC patients with a higher level of TILs and intratumoral CD8+ T cells and therefore achieve a better prognosis [77]. Intriguingly, this prognostic information can also be obtained by the combined IHC expression of TAP1 and HLA-DQA1, which can help identify patients with excellent survival rates [77].

**Figure 1:**
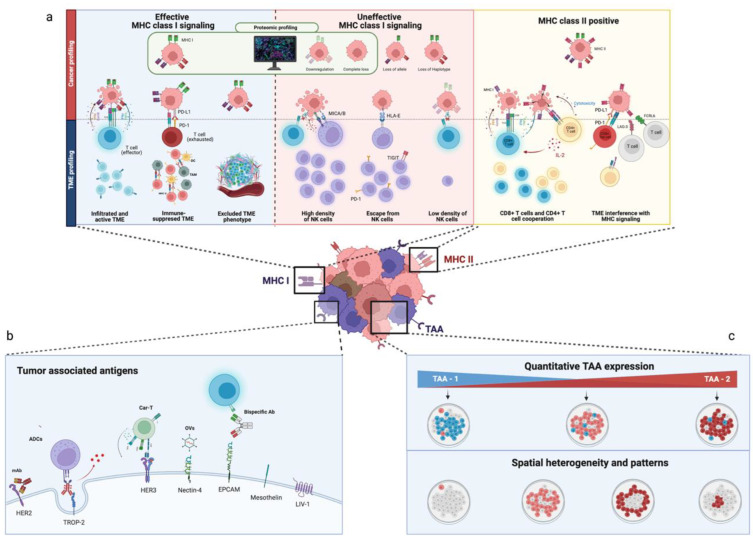
Multiplexed spatial protein profiling of breast cancer antigenicity. (**a**) Major histocompatibility complex (MHC) class I and II cancer (upper quadrants) and TME profiling (lower quadrant). MHC class I alterations that can be accurately characterized by proteomic profiling are underlined in green. In the blue box: Cancer cells with effective MHC-I signaling can be killed by effector cells or survive despite MHC-I expression if, for example, T cells are present but exhausted, or the TME lacks T cell infiltration, as is the case in an immune-excluded TME. In the green box: Cancer cells lacking MHC class I can be killed by NK cells, but can escape immune-killing in a TME that lacks NK cell infiltration or employs countermeasures that limit NK cell-mediated killing, such as in HLA-E expression. In the yellow box: MHC class II cancer cells can be killed by the coordinated effort of both CD8+ T cells and CD4+ T cells. Nonetheless, an infiltration of LAG3+ and FCRL6+ TILs can interfere with MHC class II signaling and induce cancer cell survival. (**b**,**c**) Cancer profiling of tumor-associated antigens’ (TAAs) quantitative expression and spatial distribution. Figure 1b shows relevant TAAs that can be characterized through multiplexed in situ protein profiling and some therapeutics that could benefit from TAA-profiling; Figure 1c underlines the ability of multiplexed spatial protein profiling in evaluating the combined quantitative expression of different TAAs (e.g., TAA-1 and TAA-2) in the same sample and their spatial heterogeneity. Created with BioRender.com.

Key sensitivity and resistance phenotypes related to MHC-I and MHC-II expression are summarized in Figure 1a.

### 2.2. Tumor-Associated Antigens

Tumor-associated antigens (TAA) are otherwise normal proteins, such as oncofetal antigens or surface proteins (some example surface proteins are HER2, HER3, or TROP2), which are more frequently expressed by cancer cells.

Proteomic profiling of key TAA has already proven informative, as it has formed the basis for the development of monoclonal antibodies (mAb), which are centered around the interference with some critical membrane oncogenic signaling, such as HER2-receptor overexpression. Still, preclinical and clinical data on novel therapeutics, such as antibody drug-conjugates (ADCs), bispecific antibodies, oncolytic viruses (OVs), and CAR-Ts, suggest how their efficacy can be in some cases untied from their target biological role and instead bound just to the presence of TAA. This is particularly relevant in the context of ADCs [93]. For example, trastuzumab deruxtecan (TDxD) has recently demonstrated a clinically relevant benefit in BC patients with HER2-low receptor status [94], defined as HER2 1+, 2+/ISH negative, and even as HER2-null [95]. Additionally, exploratory analysis from pivotal trials underlines how the activity of some ADCs can be influenced not only by the degree of TAAs expression in cancer cells [95,96,97,98], but also by their pattern of expression [95,99] and spatial distribution [95]. Regarding the latter, spatial TME profiling of patients treated with TDxD has recently uncovered how responders were characterized by a higher clustering of cancer cells expressing HER2 when compared to non-responders—in which HER2-expressing cells tended to be located at a great distance from each other [95]. Therefore, multiplexed in situ spatial protein profiling, by providing a higher level of accuracy and reproducibility in evaluating protein-expression levels [100], could be the optimal assay to define a proper cutoff for sensibility for those drugs whose efficacy is dependent on TAA -expression levels and spatial distribution, as depicted in Figure 1b,c.

Notably, the activity of therapeutics can be further modulated by the immune and stromal compartment. For example, the Fc region of mAbs and ADCs constitutes an interface with the immune system which can induce an antibody-dependent cell-mediated cytotoxicity (ADCC), antibody-dependent cellular phagocytosis (ADCP), and complement-dependent cytotoxicity (CDC), all of which can contribute to their efficacy [101,102]. Therefore, in view of the many combinatorial immune strategies currently in development [103,104], multiplexed spatial profiling could allow studies to further evaluate the immune–stromal contributions to drug efficacy.

## 3. Adjuvanticity

Immature APCs do not express the costimulatory receptors necessary to effectively prime T cells, which is a redundancy selected in order to protect the organism from unwanted immune activations [105]. The adjuvanticity of TME resides into the capacity to adequately permit APCs maturation, which is a process named licensing [106].

**Figure 2:**
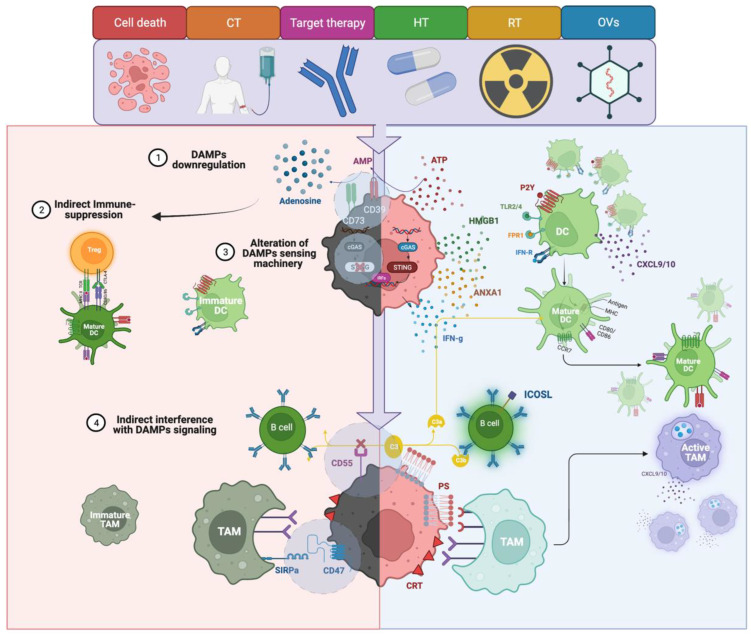
Multiplexed spatial protein profiling of adjuvanticity patterns in breast cancer. On the right side: The positive effects of regulated cell death and damage-associated molecular patterns (DAMPs) released in recruiting and activating dendritic cells (DCs), and tumor-associated macrophages (TAMs), in the tumor microenvironment (TME); further, phosphatidylserine (PS)-mediated activation of the complement system can stimulate B cells expression of ICOSL. On the left side: Some of the critical mechanisms of DAMP interference: (**1**) DAMPs’ downregulation: ATP concentrations can be limited by CD39/CD73 coordinated action, which can limit both ATP-induced adjuvanticity and favor adenosine-mediated immunosuppression; (**2**) indirect immune suppression: an indirect mechanism can interact with an effective priming, such as FOXP3+ regulatory T cell (Treg) differentiation, which can interfere with mature DC migration to tumor-draining lymph nodes (see DCs section); (**3**) alteration of DAMPs sensing machinery: STING downregulation can impair the sensing of free cytoplasmic DNA and thus STING-mediated IFN-γ signaling; (**4**) indirect interferences with DAMP signaling: CD47 expression can inhibit TAMs-mediated phagocytosis and therefore indirectly limit calreticulin and phosphatidylserine (PS) activity. CD55 can interfere with PS-induced complement-dependent cytotoxicity (CDC) and CDC-induced B cell differentiation into ICOSL+ B cells. Created with BioRender.com.

The immune system acknowledges the presence of cancer cells thanks to the recognition of warning signals associated with cell stress and death, which is more specifically named damage-associate molecular patterns (DAMPs) [107]. As cancer treatments act as cell stressors, their damage can converge in various forms of regulated cell death associated with DAMPs release. This process, labeled immunogenic-cell death (ICD) [106,108], can stimulate immune-cell recruitment and activation, and could contribute to the clinical activity of many therapeutics [5,109,110]. DAMPs can be expressed directly on the cell surface, such as phosphatidylserine (PS) and Calreticulin (CRT), or released inside the cell cytoplasm, such as free DNA/RNA, or in the TME, such as ATP, ANXA1, or HMGB1. The presence of mechanisms counterbalancing DAMPs’ immune-stimulating properties or the alteration of DAMPs’ sensing machinery, such as a reduced expression of cytosolic DNA-sensing stimulator of interferon genes (STING) [111], are powerful immune escape strategies [112], some of which can be characterized by proteomic profiling, as underlined in Figure 2.

As an example, PS-induced CDC can be limited by the expression of CD55 in cancer cells [113]. Furthermore, in BC, CD55 expression can negatively influence the complement-mediated expansion of ICOSL+ B cells, whose enrichment is associated with improved prognosis and higher responses to CT [114]. DAMPs expressed on the surface of cancer cells are a potent stimulus for their phagocytosis via tumor-associated macrophages (TAMs). However, TAMs’ final decision in phagocyting a cell is based on the balance between stimulating signals and negative phagocytic checkpoints, such as in CD47 [115]. The overexpression of CD47 can limit DAMPs-induced phagocytosis and, in BC, is associated with the development of resistance to CT [116], anti-HER2 treatments [117], and RT [118]. Consistently, only the combined IHC evaluation of TAMs’ density, and CD47 expression and not their single-marker assessment, can provide a significant prognostic value in BC [119]. ATP is a powerful immune-stimulating DAMP whose concentrations are finely regulated through the action of two ectonucleotidases, CD39, which hydrolyzes ATP in AMP, and CD73, which then converts AMP into adenosine, which is a strong immune-suppressive signal. Protein and mRNA expression of CD73 in BC correlates with worse prognoses [116,120,121,122] and treatment-resistance [123,124,125]. In TNBC, high CD73 expression levels on epithelial cells, evaluated through multiplexed immunofluorescence (mIF), is associated with reduced DFS and overall survival (OS) and lower sTILs infiltration [122]; furthermore, high CD73 expression can stratify patients with a poor prognosis despite having high TILs [122]. Interestingly, pre-clinical data suggest that chemo-resistant TNBC cells can acquire a highly immune-elusive CD73, CD47, and PD-L1 positive phenotype [116]; this further underlines the need for a multi-parametric biomarker assessment.

## 4. Patterns of Resistance to Innate Immunity 

### 4.1. Dendritic Cells

Upon antigen encounter and activation by DAMP signaling or CD40L, mature dendritic cells (DCs) express costimulatory molecules and migrate to tumor-draining lymph nodes (TDLN), where they engage with T cells to amplify the immune response [126]. In the TME, DCs, by secreting chemokines, can stimulate both the recruitment of T cells and sustain their viability [127].

DCs are classically divided into cDC1, which are responsible for CD8+ T cells priming, as well as cDC2, which engage mainly with CD4+ T cells [128]. Cancer cells can interfere in many ways with DCs’ functions as they can limit their intratumoral recruitment activation or the expression of costimulatory receptors [129], or even induce the upregulation of inhibitory proteins such as PD-L1 [130]. cDC1s intratumoral recruitment can be enhanced by NK cells, which can stimulate both cDC1 ingress into the TME and then also cluster together to foster their reciprocal functions [129,131]. BC cells can inhibit this relationship by impairing both NK cells’ survival, activity, and downregulating cDC1 expression in chemokine receptors [129]. Cancer cells can further alter DCs’ trafficking to TDLN by stimulating Treg-mediated targeting of migrating CCR7+ cDC1 [132] or by inducing an immune-suppressive local microenvironment in TDLNs [133]. This is a highly effective immune escape mechanism, as the cDC1 continuous migration to TDLN is critical in maintaining a pool of stable and proliferative stem-like TCF1+ CD8+ T cells [133,134]. This pool of stem-like cells is the precursor of effector intratumoral CD8+ T cells, which can then migrate to the tumor bed to replenish the effector T cells compartment [133]. In a preclinical work, PD-L1 expression on DCs negatively modulated the expansion of TCF1+ stem-like T cells, which is a process that was reverted by blocking PD-L1 on DCs with subsequent stem-like T-cells migration inside the tumor bed [135]. DCs could bypass this blockade by mediating a local antigen via the priming of T cells, which can be amplified in the context of tertiary-lymphoid structures (TLS); further, DCs also contribute to the scaffold of mature and functional TLS and can further enhance, in the form of follicular DCs (FDC), tumoral antigen encounters in the germinal center (GC) of functional TLS [136,137]. Additionally, in cancer nests, DCs can engage with exhausted T cells to boost their effector function when exposed to ICIs [138].

As DCs constitute a heterogeneous population of cells involved in spatially complex dynamics in the TME, with an emerging role in regulating ICI response [139], integrating multi-omics profiling with critical DC physical cell–cell interactions, niches, and migratory pathways across the TME will be essential to unveil the mechanisms that can limit DCs’ functionality.

### 4.2. Macrophages 

Contextually, macrophages can exert both immune-suppressive and stimulating functions [140]. TAMs, the most abundant immune cells in BC [22], are classically identified by surface expression in CD68 and thus subdivided into M2-TAMs, CD163+, CD204+, or CD206 +. TAMs are generally believed to be associated with an immune-suppressive function [141,142], as are M1-TAMs, CD80+ or CD86+, which can also retain some anti-tumoral and phagocytic properties. Nevertheless, although TAMs’ presence is generally considered detrimental [143], employing this M1-M2 dichotomized view has yielded many contradictory results. In TNBC, PD-L1 expression in TAMs is associated with a better OS [144] and a higher pCR rate to neoadjuvant ICI [83,145]; further, in HR+/HER2− BC patients, a higher intratumoral density of CD163+ M2-TAMs is enriched in responders that have NACT combined with ICI and hormone therapy (HT) [23]. The tumoral beds of patients with inflammatory BC (IBC) and achieving pCR tend to be more infiltrated by CD163+ TAMs; however, in non-responders, TAMs can be found closer to cancer cells and CD8+ T cells [146]. Conversely, high interactions between T cells and TAMs correlates with a pCR to neoadjuvant talazoparib in BRCA-mutated BC [147]. At the same time, in brain metastasis from HR+/HER2− BC and TNBC, a high density of intra-tumoral CD163+ M2-polarized microglia/TAMs is associated with a worse prognosis [148]. These observations highlight how, despite this M1-M2 over-simplification, TAMs’ phenotype represents a continuum of plastic states [39] that are characterized by a high degree of transcriptional diversity [149,150] and context dependency [151,152]. In fact, TAMs’ spatial and temporal adaptions vary according to tissue territories. For example, FOLR2+ TAMs, the main macrophage population in normal breast tissue, tend to be located mainly in the tumoral stroma, where they can colocalize with T cells [153] in perivascular niches [154]. Although they exhibit a lower phagocytic activity than other TAM subsets, their presence in BC is associated with a more potent T cell activation [153] and better prognosis [154]. On the contrary, TREM2+ TAMs can be found near BC cells, inside tumor nests or at the invasive margin [154], where they are associated with a dysfunctional T cell compartment and with a worse overall prognosis [153].

Additionally, the biological significance of TAMs’ interactions can vary according to the metastatic sites. For example, CD163+ M2-polarized microglia/macrophage interaction with T cells in brain metastasis is associated with a better outcome in TNBC and HR+/HER2− BC [148]. On the contrary, in a preclinical hepatic metastasis model, TAMs could induce a FAS-L-mediated direct killing of nearby CD8+ T cells [155], an interaction which is associated with a significant systemic depletion of T cells that correlate with reduced intratumoral cancer-cell specific T cells clones and T cell diversity—as was evaluated in a cohort that included patients with BC [155]. Remarkably, this mechanism could explain the worse prognosis that is demonstrated by patients with liver metastasis that is treated with ICIs [155,156].

Considering this intricate web of interactions, going beyond the M1–M2 oversimplistic characterization and achieving an extensive, spatially resolved single-cell TAMs profiling, coupled with cell profiling of other immune components such as CD8+ T cells, will be needed in order to untangle TAMs’ functional status and its influence on immune responses.

### 4.3. NK Cells

NK cells are a heterogeneous population of innate immune cells, commonly identified by the surface expression of CD56 and specialized in the direct killing of cells. In general, NK cells’ infiltration is associated with a favorable clinical outcome in many cancers [157], including TNBC-operated patients who did not receive CT [158]. In addition, NK cells’ density is associated with a higher CT sensitivity in IBC [146].

NK cells’ killing capacity is tightly regulated by the interaction with both its activating, such as NKG2D [159], and inhibitory molecules that have costimulating receptors (such as MHC class I chain-related polypeptide (MICA) A and B) and co-inhibitory receptors [160] expressed on the surface of cancer cells. As MHC I is one of the main coinhibitory receptors, cancer cells with MHC-I loss are rapidly recognized by NK cells [161] and, accordingly, show a higher presence of NK cells coupled with low infiltration of CD8+ T cells [78]. Consequently, cancer cells must acquire specific mechanisms to counterbalance NK cells killing, by reducing the engagement with stimulating receptors, as induced by membrane-shedding of MICA/B [162], or by stimulating the expression of inhibitory molecules. Regarding the latter, HLA-G expression, a nonclassical MHC-I molecule frequently expressed by BC [163], can engage the KIR2DL4 receptor expressed in NK cells; this can limit trastuzumab-mediated killing in HER2+ BC, while also inducing the expression of PD-1 on the NK cells’ surface, which is a resistance mechanism that could render them susceptible to PD-L1-mediated immune suppression [149].

In addition, NK cells exert an essential role in metastasis control, which can be influenced by the metastatic site [164,165]; for example, in liver metastasis, NK cells can sustain the dormancy of BC cells through IFN signaling [166], an antitumoral mechanism that can unfortunately be reverted by hepatic-stellate cells through the secretion of CXCL12 [166].

Cancer cells employ different mechanisms to limit CD8+ T cell-mediated killing which can inherently contribute to ICI-resistance. As those strategies could render cancer cells sensible to NK cells mediating killing, fostering NK cells in combinatorial treatments could represent a promising strategy to overcome some of the resistance nodes to ICIs. Accordingly, this will likely require a deeper profiling of the resistance mechanisms to NK cells mediated killing and the largely unexplored spatial determinants of their cancer-control. 

## 5. Homing and Migration

### 5.1. Endothelial Cells 

Through the secretion of VEGF, cancer cells can shape the phenotype of endothelial cells (EC) to achieve an optimal influx of oxygen and nutrients. However, this can induce a rearrangement of the tumor vasculature, which can exert profound immune suppression, limit the efficacy of treatments, and therefore constitutes the rationale for antiangiogenetic therapy [167]. Furthermore, as T cells homing in tissues requires their passage through EC, tumor-associated EC (TA-ECs) can therefore facilitate or constrain T cell entry [168]. As an example, they can exclude CD8+ T cells by inducing the endothelial expression of endothelin B [169] or they can directly kill them through FAS-L expression, therefore allowing, at the same time, the passage of T regulatory cells (Treg) [170]. Indeed, TA-ECs can stimulate immune recruitment through the expression of various adhesion molecules, the secretion of chemokines such as CXCL10, or even act as APCs by expressing MHC II [171].

Furthermore, TA-ECs can form highly specialized structures for the facilitating of T cell entry. These structures are more specifically named high endothelial venules (HEVs) [172] and are commonly found inside lymph nodes [173] and TLS [136]. HEVs’ density is a favorable prognostic factor and is predictive of a pCR in eTNBC [174]. Recently, Asrir et al. combined mIF, transcriptomic analysis, flow cytometry, and in vivo microscopy in order to identify a subset of TA-HEVs as the privileged portal for TCF1+ stem-like T cells [175]. They also showed that the maturation of these structures is positively regulated by ICI and is associated with ICIs’ benefit in melanoma patients [175].

### 5.2. Cancer-Associated Fibroblasts

Cancer-associated fibroblasts (CAF) constitute a highly heterogeneous population of cells that are characterized by complex transcriptional and proteomic phenotypes; they also possess many context-dependent functional states [40,176]. In fact, through single-cell profiling, four subtypes of CAFs were isolated in BC (S1 to S4), which can be defined by combining six markers (i.e., FAP, CD29, αSMA, FSP1, PDGFRβ, and CAV1) [177]. In BC, CAF-S4 presence correlates with worse OS and the development of liver metastasis [178]; further, CAF-S1 is associated with reduced infiltration of CD8+ cells, a significant attraction of FOXP3+ CD4+ regulatory T cells [177,179], and the expression of CD73, which could mediate its immunosuppressive activity [179]. Interestingly, metastatic lymph nodes are enriched with CAF-S1 and S4, where they can promote the onset of distant metastasis through CXCL12-mediated chemo-attraction and EMT-induction of CXCR4-expressing cancer cells [178].

As T cells can move around the TME just by slipping through the matrix interfibrillar spaces [180], via modulating ECM stiffness and composition, CAFs can limit TILs ingress [180], activity [181], and spatial distribution [22,47,48,182]. In patients with BC, aSMA+ CAFs can promote, through CXCR4 signaling, a fibrotic and desmoplastic TME that is characterized by a reduced T cell infiltration. Additionally, it is a phenotype that can be both reverted with CXCR4-inhibitors and can synergize with ICIs in BC mouse models [183]. Cancer cells can further support this ECM-mediated exclusion by expressing discoidin domain receptor 1 (DDR1), a collagen receptor that can promote fiber alignment and reinforce the ECM barrier [184]. In a mouse model of TNBC, high expression of DDR1 at the tumor border correlated with low intratumoral T cells, while an anti-DDR1 mAb could intensify the immune infiltration [184]. Even when T cells are allowed to reach the tumor bed, CAF can still limit, in many ways, their effector function [182]. This is because they can physically shield cancer cells [185] and form niches with quiescent cancer cells (QCCs) and dysfunctional dendritic cells, which are reservoirs that can limit T cell-mediated killing and undermine immunotherapy efficacy [186]. Furthermore, they can act as a decoy through the expression of MHC class I, engage with CD8 T cells, and then induce their killing through FAS-L mediated signaling [187].

Notably, CAFs’ biological function seems to be influenced by the BC subtype. For example, in ER+ BC, a high number of fibroblasts are only associated with a worse prognosis when not accompanied by high TILs, whereas patients with high TILs, and high spatial interactions, with fibroblasts have an excellent prognosis [188].

Coupling multiplexed CAFs’ phenotype characterization—through surface biomarkers, spatial distribution, and their contribution to ECM modulation with techniques capable of capturing TME’s stiffness, solid stress, and fluid pressures—will be instrumental in understanding the physical traits of cancer [189] and their influence on immune resistance mechanisms.

### 5.3. Chemokines

The regulation of cell behavior, spatial localization, and different interactions that are mediated by chemokine and cytokine signaling [190] can be explored by the use of spatially resolved transcriptomic profiling of selected chemokines, coupled with a proteomic characterization of their specific receptors or key cell target. For example, in melanoma, by combining spatial proteomics and transcriptomics, authors identified tumor patches that are characterized by a high transcriptomic expression of CXCL9 and CXCL10 (which are key chemokines for T cell recruiting) coupled with dysfunctional T cells expressing CXCL13 [191], a chemokine which can regulate B and T cell interactions in their assembly into TLS. Similar patches were identified in BC, where stem-like TCF1+ T cells were in close contact with CXCL10-producing cells [192]. Interestingly, by employing three-dimensional spatial imaging of TDLN, Duckworth et al. recently evaluated the importance of chemokine-induced positioning of a T cell as a determinant of their interaction with cDC1 and their differentiation in stem-like TCF1+ T cells [193].

## 6. Recognition and Killing

### 6.1. TILs Density

TILs include CD8+ T cells, CD4+ T cells, and B cells infiltrating the tumoral stroma and bed. A more comprehensive overview of the prognostic and predictive significance of TILs’ bulk density evaluation in BC goes beyond the scope of this review, for which we direct the reader to [24].

Briefly, the evaluation of TILs’ stromal density (sTILs) on hematoxylin and eosin (H&E) has demonstrated both prognostic and predictive potential in BC [24,194]. In addition, it has also achieved level 1b evidence for clinical validity in eTNBC [24,195,196] where sTILs density can identify subgroups of patients with an excellent outcome [195,197,198,199,200,201]. In residual diseases, both the presence of high sTILs [33,202] and an increase from baseline values are associated with improved DFS [202]. Strikingly, sTILs’ density is associated with improved pCR rates in relation to CT-ICI, but not to the ICI-specific response rate [203,204,205,206,207]. Nevertheless, in the GeparNuevo trial, an increase in intratumoral TILs’ density after a window-of-opportunity phase—where durvalumab was administered two weeks before nab-Paclitaxel—was predictive of a pCR exclusively in the ICI-arm [203]. In mTNBC, although sTILs’ overall density tends to be generally lower [208], it is still associated with an improved survival [206] and response to ICIs [109,209,210,211,212].

In HER2+ eBC, sTILs are predictive of trastuzumab [198,213] and pertuzumab [214] adjuvant benefits and are enriched in patients achieving a pCR [215,216,217]; counterintuitively, high sTILs in residual-disease post-NACT have been associated with lower DFS [218]. In the advanced setting, sTILs are predictive of OS from first-line therapy with the administration of pertuzumab, trastuzumab, and docetaxel [219] and from exploratory analysis from trials that combine anti-HER2 treatments with ICIs [220].

In HR+/HER2−BC, the significance of TILs’ presence still needs to be untangled [85]. In the neoadjuvant setting, greater TILs are associated with a higher rate of pCR, but a shorter OS and DFS, particularly in patients with suboptimal responses to CT [195]. In the adjuvant setting, on the other hand, patients with higher TILs have a worse prognosis when receiving hormone therapy alone, but better DFS when receiving CT [188,221]. Nonetheless, patients with a high infiltration of sTILs show higher rates of the pCR when treated with a combination of hormone therapy and ICIs after an induction phase with CT [23].

Notably, TILs’ measurement on H&E does not take into account the different type of lymphocytes that constitute the immune infiltrate, their phenotype, functional status, and spatial distribution. Moreover, the killing performance of TILs can be limited by many mechanisms besides a tout court limitation of their overall infiltration of the tumor bed. This highly relevant data, which could explain some of the contradictory results obtained by TILs’ bulk density evaluation, could be instead provided by multiplexed TME profiling of TILs’ subpopulations and their spatial organization.

### 6.2. Spatial Organization

The distribution of TILs within the TME show some recurrent patterns that are conserved across histologies. Tumor infiltration can be classified as hot or immune-inflamed (IN). That is, hot when the tumoral bed is highly infiltrated by immune cells; immune suppressed (IS) if the immune infiltration is present but limited by an immunosuppressive TME; immune-excluded (IE) when TILs are present at the tumor border, but are somehow not allowed to step into the tumor bed; and cold or immune-desert (ID) when there is an absent immune infiltration [222]. Multiplex spatial profiling has allowed to extensively explore those arrangements in the context of BC [22,37,43,47,48], and has been recently reviewed in [223]. Intriguingly, TILs’ density and spatial organization can also be evaluated by employing deep-learning techniques from H&E-stained tissues [224,225], thus limiting some of the analytical challenges related to TILs’ IHC evaluation [226].

The IN phenotype is typically associated with a better prognosis, a better response to CT and ICI [47,48] and it is characterized by MHC I expression, as well as high infiltration of CD8+ T cells with high TCR clonality and immune-effector properties [47], coupled with high infiltration of DCs in close contact with CD8 cells [48]. On the other hand, CD8+ T cells’ presence tends to be counterbalanced by higher FOXP3+ CD4+ T cells (Treg) and a higher expression of suppressive immune checkpoints such as LAG3, TIM-3, TIGIT, PD-L1, and CTLA-4 [47], which are potentially actionable with ICIs. The IE and ID patterns are generally associated with a worse prognosis [47,48] and ICI resistance [48]. The IE phenotype tends to be associated with ECM remodeling, fibrotic signaling, and collagen 10 deposition [48], which can all physically limit T cells. Further, this is coupled with TGFb-mediated suppression of T cell migration [227] and metabolic competition for critical resources [47,48]. ID cancers are frequently characterized by lower MHC I expression and high levels of B7-H4 [47] as well as an immunosuppressive protein that is mutually exclusive with PD-L1 [47,228].

Another example of the importance of assessing the spatial organization in the BC TME, is provided by the pattern evaluation of the expression of immune-regulatory proteins. Indeed, inflamed TME phenotypes tend to be associated with PD-L1 expression in tumor cells and PD-1 expression in CD8+ T cells [22,47]. Contrastingly, compartmentalized or excluded TMEs that are present with PD-1 positivity on CD4+ T cells and higher stromal expression of PD-L1 and IDO1 [22,47]—a phenotype which, when co-expressed on APCs, is associated with an enhanced sensibility to atezolizumab in eTNBC [83]. In this regard, spatial imaging—by providing spatially resolved, quantitative, and operator-independent high-throughput data [75,229,230]—could overcome some limitations related to the analytical challenges as well as the temporal and spatial heterogeneity dynamics of immune regulator assessment [21,31]. Indeed, multiplexed spatial imaging of immune-regulatory expression and interactions has already provided insightful prognostic information in BC [75,92,148].

Table 1 reestablishes key immune biomarker data from clinical trials that explored ICIs’ role in BC.

### 6.3. T Cell States and Trajectories 

#### 6.3.1. CD8+ T Cells

CD8+ T cells are the main effectors of immune-mediated tumor killing. Consistently, their density is a favorable prognostic factor in patients with TNBC [33,244]; further, HER2+ BC is associated with higher response rates to both NACT and, in the metastatic setting, to ICIs [208]. Contrarily, in HR+/HER2− BC, high intratumoral CD8+ T cells are associated with a resistance to tamoxifen in the adjuvant setting [245], but with higher response rates in patients treated with ICI-CT when combined with hormone therapy [23], which results in better OS in regard to brain lesions [148].

The interpretation of this data will require a more comprehensive approach [85] as CD8+ T cell density does not account for their complex functional status. In fact, CD8+ T cells constitute a continuum of phenotypes, commonly crystallized in some critical cell states across a trajectory of cell dysfunction [246,247]. CD8+ TFC1+ stem-like T cells are T cell precursors with a conserved effector function and proliferative capacity. Chronic antigen exposure turns these cells into a state of exhaustion [248]. In addition, these exhausted T cells (Tex) are in fact characterized by reduced proliferative and effector capacities, and by the expression of many immune suppressive checkpoints on their membrane, such as in PD-1, CD39, TIGIT, TIM-3, and LAG-3 [246,247].

Stem-like T cells’ plasticity [249], proliferation ability [250], and correlation with ICI-response [251,252] make them the ideal target of ICIs. Spatial profiling has shed light on the importance of their spatial positioning as a key determinant in their predictive role [253]. Stem-like T cells can, in fact, colocalize with MHC-II+ APCs in perivascular immune niches when located in the tumoral stroma [252] and with the appearance of secondary lymphatic tissue [138,253,254,255]. In kidney cancer patients, a lower number of those niches was associated with resistance to ICI [255]. Moreover, patients who relapsed showed a reduction from the baseline in the quantity of these aggregates [255]. Meanwhile, ICI exposure can increase the abundance of these T cells; further, APC aggregates and their relative richness correlate with ICI response [254].

Tissue resident-memory T cells (Trm) are a population of T cells that express a tissue-residency transcriptional program [256,257], as well as surface receptors CD39 [258,259] and CD103 [260] with cytotoxic and effector functions and the expression of inhibitory receptors, including PD-1 [261]. Their density correlates with the response to ICI in multiple cancer types [262], regardless of the number of TILs and total CD8s [263,264]. In addition, Trm population expands in responders to ICIs [265]. In HER2−/HR− BC, intratumoral Trm are associated with a better OS and DFS [41,266,267,268], even more than the total CD8+ T cell count [41,268], particularly when in close contact with cancer islands [266].

Notably, a large subset of lymphocytes infiltrating the TME are not cancer-specific [259] and are therefore labeled bystanders. As they are not antigen experienced, they do not express exhaustion markers, such as CD39 [259]. Indeed, their biological role is still not completely understood, as they constitute a heterogenous population that can express some state of activation and therefore could take part in the immune response [269]. In fact, a bulk evaluation of the biological role of CD8+ T cells could be undermined by including those potentially inactive T cells, as mIF quantification of CD8+ CD39+ subpopulations over total CD8+ cells in NSCLC can stratify responders over non-responders, regardless of total CD8+ value [270].

#### 6.3.2. CD4+ T Cells

CD4+ T cells are a plastic population of cells generally divided into two groups, non-Treg CD4+ T cells and Tregs.

Non-Treg CD4+ T cells constitute a heterogeneous population of cells [271], which can have an indirect role in cancer control by collaborating with CD8 T cells in order to enhance their killing proficiency [272]. They can also directly recognize cancer cells expressing MHC II [87] or even kill cancer cells [89], as in HR+/HER2− BC patients treated with an induction phase of CT followed by nivolumab + HT, stromal Granzyme+ CD4+ T cells were enriched, as a result, after exposure to ICI [23]. Interestingly, as for CD8+ T cells, antigen exposure can induce a state of exhaustion in CD4+ T cells, which is characterized by the expression of PD-1, CTLA-4, and CD39 [272]. The latter could be employed to isolate the subgroup of bystander CD39- CD4+ T cells, which are non-specific for cancer antigens and whose biological role is still unexplored [272].

Tregs, identified by the expression of their master transcriptional regulator FOPX3+ [273], are cells that are specialized in limiting immune damage across all tissues. Tregs can exert this function by secreting immunosuppressive cytokines, such as IL-10 and TGF-b, by stimulating the production of adenosine with CD39 [274], and expressing suppressive immune checkpoints, such as CTLA-4 and PD-1 [275,276]. Despite this outstanding immunosuppressive arsenal, the evaluation of Treg has provided some conflicting results, particularly in BC [194,277]. In HR+ BC, FOXP3+ CD4+ TILs were associated with higher grade cancers, lymph nodes involvement, and worse prognoses; however, it must be noted that this prognostic significance was lost in multivariate analysis [278]. In TNBC, higher numbers of FOXP3+ T cells correlate with better survival [279], but this was irrelevant in patients with low CD8+ TILs [280], as CD8+ T cell presence is generally counterbalanced by a high Treg infiltrate [47]. Nonetheless, in patients with eTNBC, the addition of FOXP3+ Tregs evaluation to a multiparameter model provided relevant prognostic information, beyond classical clinical–pathological factors and TILs [33].

Tregs finely tune their population through sophisticated sensing strategies and highly specialized multi-cellular modules [281,282,283], which further underline the spatial determinants of Tregs’ function. In fact, in lymph nodes, Tregs can recognize activated CD4+ T cells, form tightly packed aggregates, and, by surrounding them, compete with survival signaling and physically constrain their expansion [283]. Alternatively, Tregs’ expression of CTLA-4 can interact with CD80/86, which is expressed on activated migrating DCs. Further, this can both stimulate Treg expansion and induce a transendocytosis of CD80/CD86 from the DC membrane [132], therefore limiting any further T cell priming [282]. Significantly, as CD4+ T cells can express PD-1 [22], particularly in compartmentalized TME phenotypes, the anti-PD-1/PD-L1s targeting of Tregs could enhance their expansion and therefore be responsible for anti-PD1 resistance [284]. This is underlined in gastric cancer patients treated with anti-PD1 ICI and who are experiencing progressive diseases [285]. Furthermore, the expression of PD-1 is particularly relevant in regulating the activation status of TLS via the balancing of the interaction between PD-1+ CXCR5+ CD25+ FOXP3+ follicular regulatory T (Tfr) cells and follicular helper T (Tfh) cells [136,286].

#### 6.3.3. TIL-B and TLS

B cells are plastic cells with multiple phenotypes [287] that can help coordinate the anti-tumor immune response through the production of cytokines, the presentation of antigens to T cells, and the production of antibodies against tumor antigens [288], whose humoral action can help mediate ADCC, ADCP, and CDC by cooperating with many stromal cells [287].

In BC, an enrichment in TIL-B is a favorable prognostic factor [146,174,289,290,291,292,293], particularly when located inside cancer islands [294], and is associated with higher response rates to CT [295,296,297], anti-HER2 agents [298] in HER2+ BC, and ICIs in eTNBC [83,207]. Nonetheless, B cells’ biological role seems to be highly dependent on their interaction with other cellular components of TME [287]. In fact, the formation of intratumoral B-T cell clusters undergoing cell receptor-driven activation, proliferation, and IgG-isotype switching, is associated with better outcomes in patients with TNBC [290]. However, cell phenotyping is critical, as some B cells can express a regulatory phenotype (Breg) associated with IL-10 expression and immunosuppressive properties. Accordingly, Tfr and Breg interaction can generate a highly immunosuppressive phenotype that correlates with short metastases-free survival (MFS) in BC [299].

The highest degree of B-T cell associations is the formation of TLS, which are organized lymphoid structures that resemble secondary lymphoid tissues, whose density correlates with ICIs benefit in numerous tumor types [300,301,302,303] and are associated with favorable prognostic [304] and predictive value for CT responses in BC [291]. TLS possess various degree of maturation, as immature TLS are composed of a T cell zone and a B cell zone without a germinal center (GC) [136]; meanwhile, mature and active TLS are characterized by a GC coupled with Tfh cells in the T cell zone and mature DC [136]. Defining these TLS states, which can be achieved with multiplex profiling, is relevant, as patients with more than two active TLS experience a better DFS in BC [136]. Importantly, TLS functional status seems to be influenced by the interaction between PD-1+ Tfh cells and Tfr cells [136,286,305], a balance of which ICIs could tip in favor of Tfh and thus help generate a more effective immune response. Interestingly, natural antibodies could be one of the main outputs of those active TLS. Through combined spatial proteomic and transcriptomic profiling of renal cancer, B cells were seen to mature into plasma cells when inside TLS and, in turn, migrate inside tumor beds along CAF tracks [306]; furthermore, the authors detected macrophages in close proximity to apoptotic IgG-stained tumor cells and found a correlation between the percentage of IgG-coated tumor cells and, therefore, a higher response to ICIs.

**Figure 3:**
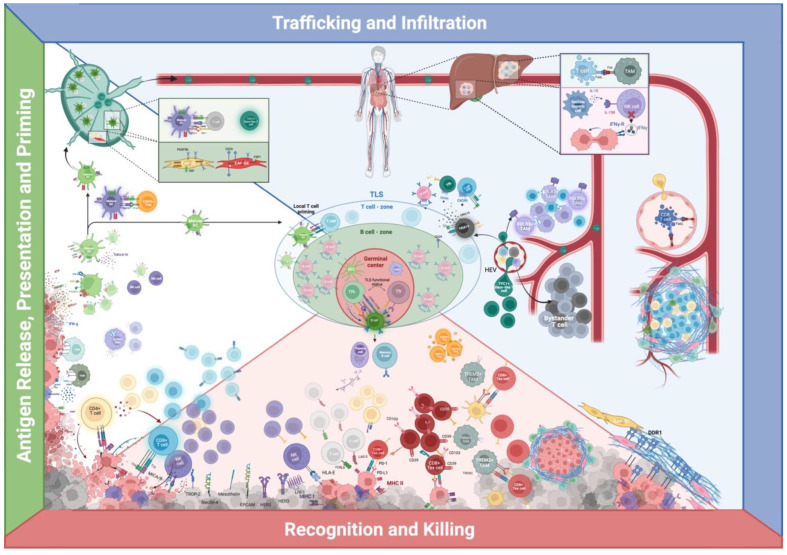
The cancer-immunity cycle and multiplexed in situ spatial protein profiling of relevant cellular interactions and niches in breast cancer. Green—antigen release, presentation and priming: tumor-associated antigens (TAAs) are captured by innate immunity, whose activation is enhanced by damage-associated molecular patterns (DAMPs) and limited by mechanisms altering TME’s adjuvanticity, as depicted in Figure 2. Activated dendritic cells (DCs) can therefore migrate to tumor-draining lymph nodes (TDLN), where they can prime naïve T cells. Regulatory FOXP3+ T cells (Treg) can target migrating DCs and thus limit T cell priming. NK cells can favor DCs’ recruitment and physically interact with DCs and, in TDLN, DCs’ interaction with T cells can foster a transiently activated T cell phenotype or stimulate a stem-like T cell phenotype. Different subtypes of cancer-associated fibroblasts (CAFs) can populate TDLN and influence the incidence and pattern of metastasis, as CAF-S1 and S4. DCs can prime T cells in tertiary lymphoid structures (TLS), thus bypassing TDLN. Blue—trafficking and infiltration: cellular components of the immune system must access and repopulate the TME to exert cancer-immune control. On the far right: cancer cells can limit CD8+ T cells’ endothelial access by inducing the expression of FASL on tumor-associated endothelial cells (TA-EC), meanwhile positively regulating regulatory FOXP3+ T cell (Treg) passage. Cancer cells can alter T cells intrastromal motility by directly altering the extracellular matrix (ECM)—such as with DDR1 expression—or indirectly modulate ECM-structure by subjugating different CAFs subpopulations. On the right: CD8+ T cells engage in peri-vascular niches with FOLR2+ tumor-associated macrophages (TAMs); TCF+ stem-cell like T cells can access the TME through specialized high endothelial venules (HEVs). Further, in grey, the bystander T cells, whose role in BC is still unclear. In the middle—human figure: TME profiling of metastatic sites. > On the upper portion: Cells can indirectly limit T cell infiltration by systemically depleting T cells through TAMs-mediated siphoning of tumor-specific CD8+ T cells in hepatic metastasis. > On the lower portion: hepatic stellate cells’ interaction with NK cells can limit their cancer control ability. TLS present the unique potential of locally stimulating both T cell priming and activation, B cell maturation, and natural antibody production. CXCL13+ follicular T cell helpers (TfhX13) can coordinate CXCR5 positive immune cells into aggregating in multi-cellular structures, which could be the precursors of TLS. Red—recognition and killing: innate and acquired immune cells can find and destroy cancer cells by leveraging their antigenicity (MHC class I/II expression), which is counterbalanced by many immune-evasive mechanisms described in the antigenicity section in Figure 1. Even when T cells are allowed to reach the tumoral bed, cancer cells can express PD-L1 and directly limit the activity of T cells expressing PD-1 or hide from them in niches constructed by CAFs; furthermore, T cells can be physically surrounded by TREM2+ TAMs and other APCs, which can alter their killing endeavor. Created with BioRender.com.

Figure 3 recapitulates multiplexed in situ spatial protein profiling of some relevant cellular interactions and niches from the perspective of the cancer immunity-cycle.

## 7. Conclusions

Spatial biology has just begun to unveil the concealed sets of rules that regulate cell dynamic interactions and multicellular organization in BC. Although single-cell technologies have identified the letters of this language, ultimately, heterogenic cells and plastic phenotypes seem to be strictly dependent on their relationship with their surroundings. In this review, we underlined the outstanding potential of multiplexed single-cell spatially resolved epitope colocalization in defining cellular phenotypes and functional status, which can be achieved by computing their reciprocal spatial coordinates into a precise tissue cartography. Although more sophisticated, yet time-consuming and resource-demanding, technologies are being developed to achieve an extensive TME profiling, multiplexed spatial protein profiling could help translate into clinical practice some of the biological information that is inferred from those more complex analyses through a rationally selected panel of biomarkers. Still, the clinical application of this approach will require to overcome many analytical challenges related to the pathological workflow. These challenges entail panel assembly and standardization, as well as the computational-intensive workload required to analyze the massive datasets generated by this high-throughput image analysis. Furthermore, as many means of communication, other than the proteomic, contribute to modulating the response to therapeutics, deep multi-omic TME profiling will be crucial to identify the strategies that cancer cells have put in place to escape immune control at the patient level, therefore guiding combinatorial strategies while reducing the burden of ineffective treatment.

## Figures and Tables

**Table 1:** Main clinical trials exploring ICIs’ role in BC.

BC Subtype		Trial	Phase	Treatment Arms	Primary Efficacy Endpoints	Biomarker Analysis	*N*. Patients (Trial Status)	References
**TNBC**	**Early** **TNBC**	Keynote-173	I/II	Pembro + T +/− Cb > AC− > S− > pembro adj x 1yr	pCR	PT: sTILs and PD-L1 associated with pCR and ORR; and OT: sTILs associated with pCR and ORR	60 (completed)	[205]
		I-SPY 2	II	Pembro/placebo + T > AC	pCR	MHC II expression predictive of response to ICI	64 (completed)	[82]
		NeoPACT	II	Pembro + CbD	pCR	High sTILs are associated with higher pCR	117 (active, not recruiting)	[204]
		Keynote-522	III	Pembro/Placebo + CbT > AC > CH > pembro adj x 1yr	pCR + EFS	PD-L1 CPS not predictive of response to ICI	602 (completed	[12]
		NeoTRIPaPDL1	III	Atezo/placebo + NabP + Cb > S > anthracycline-based CT	EFS	pCR rate + 10% to atezo in immune-rich TME (PDL1 IC+, high/intermediate sTILs/iTILs) High expression of GATA3 and CD20, epithelial of HLA-DR and Ki67 in both epithelial and TME favors atezo arm; and PD-L1 + IDO+ APC and CD56 NE cells were associated with a higher response rate to atezo.	278 (active, not recruiting)	[83,206]
		Impassion031	III	Atezo/placebo + NabT > AC	pCR	PT: PD-L1 IC+ and TC+, sTILs, iTILs, and TLS linked to improved pCR in placebo arm; and OT: numerical increase in iTILs and PD-L1 in immune cells in patients with pCR in ICI arm; further, ICI can promote close contact of TILs to tumor nests	455 (active, not recruiting)	[207]
		NCT02489448	I/II	Durva + nab-paclitaxel > AC	pCR	IHC: sTILs associated with higher pCR, sTILs, and PD-L1 do not predict benefit in multivariate analysis; MHC II expression predicts response to ICI; and mIF: PD-L1 TC+, IC+ in the stroma and PD-L1+ CD68+ TAM compartment each associated with higher rates of pCR	69 (completed)	[82,231]
		GeparNuevo	II	Durva/placebo + nab-paclitaxel	pCR	PT: High sTILs associated with higher pCR in both arms; OT: iTILs increase = higher pCR in ICI arm; and RD: high TILs associated low rates of relapse in both arms	174 (completed)	[203,232,233,234]
	**Metastatic** **TNBC**	Keynote-119	III	Pembro/CT	OS	sTILs associated with ICI benefit, in particular in previously untreated mTNBC; and PD-L1 TC+ adds predictive power to pembro arm	622 (completed)	[26,209,210]
		Keynote-086	II	Pembro	DCR, ORR, DoR, PFS, OS	sTILs, PD-L1 CPS+, and CD8 IHC evaluation correlate with the response rate to pembro	254 (completed)	[211]
		Keynote-355	III	Pembro/placebo + NabP/T/Gem + Cb	PFS, OS	PD-L1 CPS ≥ 10 correlates with improved PFS and OS	847 (active, not recruiting)	[11]
		ENHANCE-I	Ib/II	Pembro + eribulin	ORR	PD-L1 numerically higher ORR	167 (completed)	[235]
		Impassion130	III	Atezo/placebo + NabT	PFS, OS	PD-L1 IC+ predictive of ICI benefit; and PD-L1 IC+ and either PD-L1 TC+ or 10% or more sTILs had the highest clinical activity with A + nP	902 (completed)	[10,212]
		Impassion131	III	Atezo/placebo + T	PFS	PD-L1 IC+ does not predict benefit	651 (active, not recruiting)	[28,236]
		TONIC	II	Nivo/nivo after induction with CT or RT	PFS	PD-L1 IC+, sTILs, and CD8+ higher in responders	67 (active, not recruiting)	[109]
**HER2**	**Early HER2+ BC**	Impassion-050	III	Atezo/placebo + THP + AC > S > atezo/placebo + HP	pCR	PD-L1 IC+ does not predict pCR	454 (active, not recruiting)	[237]
	**LABC/Metastatic** **HER2+ BC**	NCT02605915	Ib	In LABC: Atezo + HP or atezo/T-DM1 > THP + Cb In mBC: Atezo with trastuzumab/pertuzumab, atezo with T-DM1, or atezo with THP	ORR + DoR	PT: No correlation between response and PD-L1 IC+, TC+, sTILs, and CD8+ T-cell density in central tumor area and immune phenotypes (ID, IE, or IN); OT: increase in PD-L1 IC+ in both cohorts, no association with response; and LABC: significant increase in CD8+ T cells density in the central tumoral area, but not correlated with pCR, no increase in mBC	76 (completed)	[238]
	**Metastatic** **HER2+ BC**	PANACEA	I/II	Pembro + trastuzumab	ORR	sTILs correlate with ORR and disease control, as well as higher clinical benefit in PD-L1 + CPS	58 (completed)	[239]
		KATE-2	II	Atezo/placebo + T-DM1	PFS	High CD8 T cells at invasive margins favor atezo arm in subgroup analysis	1486 (completed)	[220]
**HR+**	**Early** **HR+ BC**	GIADA	II	Nivo + exemestane + triptorelin + EC	pCR	PT: in pCR patients higher in sTILs, iTILs (iCD4, I CD8, and iCD4+ FOXP3+), and TAMs (intratumoral); TAMs: stromal CD68+ CD163+ TAMs) immune-checkpoints co-expression: PD-1+ on T cells, and PD-L1 on TAMs (CD68+ PD-L1+ and CD68+ CD163+ PD-L1) higher in pCR; OT after CT: sTILs increase, increase in CD8+ T cells, decrease in FOXP3+ CD4+ T cells, and CD68+ CD163+ TAMs; and OT after Nivo: increase in intratumoral and stromal CD8+, CD8+ Granzyme+ T cells and stromal CD4+ Granzyme B+ T cells.	43 (completed)	[23]
		ISPY-2	II	Pembro/placebo + T > AC	pCR	MHC II expression predicts response to ICI	89 (completed)	[82]
	**Metastatic HR+ BC**	Keynote-028	Ib	Pembro	ORR	sTILs do not predict PFS	83 (completed)	[240]
		KELLY	II	Pembro + eribulin	CBR	PD-L1 does not predict benefit	44 (completed)	[241]
		NCT03051659	II	Pembro + eribulin	PFS	sTILs and PD-L1 do not predict benefit	88 (active, not recruiting)	[242]
		NCT03044730	II	Pembro + capecitabine	PFS	sTILs and PD-L1 do not predict benefit	14 (completed)	[243]
		NIMBUS	II	Pembro + nivo in TMB-H	ORR	sTILs and PD-L1 do not predict benefit	20 (active, not recruiting)	[28]

PT = pre-treatment; OT = on-treatment; mBC: metastatic BC; LABC: locally advanced BC; S = surgery, CT = chemotherapy; ICI = immune checkpoint inhibitors; Atezo = atezolizumab; Durva = durva; Pembro = pembrolizumab; D = docetaxel; E = epirubicin; C = cyclophosphamide; T = taxane; Gem = gemcitabine; H = trastuzumab; P = pertuzumab; Cb = carboplatin; ORR = objective response rate; PFS = progression-free survival; OS = overall survival; pCR = pathological complete response; EFS = event-free survival; DoR = duration of response; DCR = disease control rate; CBR = clinical benefit rate; IC = immune cells; TC = tumoral cells; CPS = combined positive score; ID = immune desert; IE = immune excluded; and IN = immune inflamed.
